# Migration, personal physical safety and economic survival: drivers of risky sexual behaviour among rural–urban migrant street youth in Kampala, Uganda

**DOI:** 10.1186/s12889-022-13516-y

**Published:** 2022-06-04

**Authors:** Mulekya Francis Bwambale, Deborah Birungi, Cheryl A. Moyer, Paul Bukuluki, Bart van den Borne

**Affiliations:** 1 Medicine and Life Sciences, University of Maastricht Faculty of Health, Care and Public Health Research Institute (CAPHRI), Maastricht, Netherlands; 2 Department of Social Work and Social Administration, Makerere University School of Social Sciences, P.O. Box 22234, Kampala, Uganda; 3 Department of Population Studies, Makerere University College of Business and Management Sciences, School of Statistics & Planning, Kampala, Uganda; 4 University of Michigan Medical School, Ann Arbor, MI USA

**Keywords:** Migration, Street youth, Pathways of risky sexual behaviour

## Abstract

**Background:**

Despite the vulnerabilities associated with the youth migration process, knowledge on the drivers of risky sexual behaviour among migrant street youth is limited. This study sought to explore the pathways driving risky sexual behaviour among rural–urban migrant street youth in Kampala, Uganda.

**Methods:**

We conducted 11 focus-group discussions composed of 8–10 participants each, and 15 in-depth interviews with urban street adolescents and youth aged 12–24 years. We purposively recruited street youth who had migrated from other districts to Kampala, Uganda, and who identified themselves as street youth. Data were analysed thematically using an inductive approach facilitated by Dedoose software.

**Results:**

The migration journey acted as a catalyst for risky sexual behaviour among the adolescents and youth moving from rural districts to Kampala. Three primary pathways were found to drive risky sexual behaviour of street youth: 1) rural–urban migration itself, through sexual exploitation of and violence toward street youth especially young girls during movement, 2) economic survival through engaging in casual jobs and sex work upon arrival in the city, and 3) personal physical safety through friendships and networks, which consequently lead to having multiple sexual partners and unprotected sex. Engagement in risky sexual behaviour, especially sex work, was found to be an adaptation to the challenging and complex street life within the city.

**Conclusions:**

This study highlights the migration process, personal physical safety and economic survival as major pathways driving risky sexual behaviour among rural–urban street youth in Kampala. Interventions to improve sexual health, physical safety and protection of street youth during the migration process and within the city spaces should be prioritised.

## Introduction

In sub-Saharan Africa, street children and youth are among the most marginalised groups in society. They exhibit a high burden of disease, including due to risky sexual behaviour and drug exposures, and hence are vulnerable to poor reproductive health practices, human immunodeficiency virus and other sexually transmitted infections [1–3]. Push factors for the increasing number of children and youth on the streets include poverty, family disintegration and violence at home; while perceived freedom and a perceived better life on the street are two of the pull factors to urban street life [4, 2]. Within urban areas, street youth have been reported to experience verbal, physical and sexual abuse and violence, including risky sexual behaviour such as unprotected sexual intercourse and having multiple sexual partners [5, 6]. In addition, the deteriorating economic and living conditions in urban areas have been found to increase risky sexual behaviour, including transactional sex and having multiple partners [7, 8].

In Uganda, rural–urban migration of youth has occurred for decades, leading to an influx of young people in cities. Studies show that migration is a critical factor in high-risk sexual behaviour and that its importance varies by the direction of movement [9]. Age, home environment and gender are important correlates of migration [10]. While migration itself does not cause illness, the conditions and circumstances surrounding the migration process can create risks to the physical, mental and social well-being of migrants [11]. Individual factors and lifestyle factors, social and community influences, living and working conditions, and cultural and environmental conditions can impact the health of migrants, their families and communities [12]. Sex work has been reported to be high among urban youth living in the slums of Kampala [13] and is often used as a means to make a living, especially among women [14].

While risky sexual behaviour is common among urban and street youth [15–18], little is known about the pathways that drive risky sexual behaviour in this group, especially those with rural–urban migration experience. None of the available literature has explored pathways driving migrant street youth’s risky sexual behaviour in Uganda. Inspired by the findings of the quantitative study which found an association between street youth intra-urban mobility and involvement in sex work [19], a qualitative inquiry to establish the pathways driving risky sexual behaviour among the migrant street youth was conducted. In particular, the current study explores the role of the migration process, personal physical safety and economic survival as potential drivers of risky sexual behaviour among migrant street youth in Kampala. Understanding these pathways is essential for designing interventions to improve migrant street youth’s sexual and reproductive health, their safety and protection from sexual abuse during migration and within the city spaces. The United Nations 2030 Agenda for Sustainable Development calls on countries to overcome health inequalities, guided by the principle of ‘leave no one behind’, of which internal migrant street youth should be included. Within the Sustainable Development Goals (SDGs), migration is considered an important contributor to sustainable development [20]. In this paper, migrants were defined as street youth who migrated from other districts and regions of Uganda to Kampala, the capital city.

## Methods

This paper is based on data collected through 11 focus-group discussions (FGDs) and 15 in-depth interviews (IDIs) among migrant street youth living in Kampala, Uganda. To investigate drivers of risky sexual behaviour within the migration context, we purposively recruited street youth with rural–urban migration experience who had stayed in the city for at least three months before the study. This period was considered adequate for the migrant street youth to have settled into the new urban environment and be able to share their early experiences on migration and risky sexual behaviour within the new urban setting. Those who had stayed in the city longer were also able to provide their long-term perspectives on their migration and sexual behaviour. For migrant street youth aged below 18 years, guardian/caregiver consent was obtained. The FGDs aimed at gaining a broader understanding of street youth's migration and sexual and experiences; while the IDIs helped to unveil a deeper understanding of their sexual behaviour, personal experiences and coping mechanisms during the migration process. To enhance open discussion, participants were stratified by ethnicity, sex and age to reflect developmentally appropriate questions regarding their sexual behaviour. Table 1 summarises the study participants by age and gender.

**Table 1:** Study participants for the IDIs and FGDs by gender and age, 2019

Type of interview	Category of participant by gender	Age group (years)	Ethnic language	No. of interviews
IDI	Young males on the street	12–16	Luganda	3
	Young females on the street	12–16	Ngakarimajong	3
	Older males on the street	17–24	Luganda	4
	Older females on the street	17–24	Luganda	3
			Ngakarimajong	2
**Total**				**15**
FGD	Young males on the street	12–16	Luganda	2
	Young females on the street	12–16	Ngakarimajong	3
	Older males on the street	17–24	Luganda	2
			Ngakarimajong	2
	Older females on the street	17–24	Luganda	1
			Ngakarimajong	1
**Total**				**11**

A team of eight (three male and five female) trained research assistants conducted the interviews using two local languages, namely *Luganda* and *Ngakarimajong*. Since many street youth, especially of Karamojong origin, were not fluent in *Luganda,* which is commonly spoken in the city, the study engaged *Ngakarimajong* native speakers to conduct the interviews with this subgroup. The interviewers had a minimum of a bachelor’s degree in social sciences, had previous experience in qualitative research and had participated in the quantitative survey that preceded this study. Furthermore, the interviewers were mainly youth and familiar with the study area and street youth. These attributes enabled them to build a strong rapport with the participants. In addition, some of the interviewers had rural and urban life experience, whilst others were recruited from districts where some of the street youth had migrated.

**Fig. 1:**
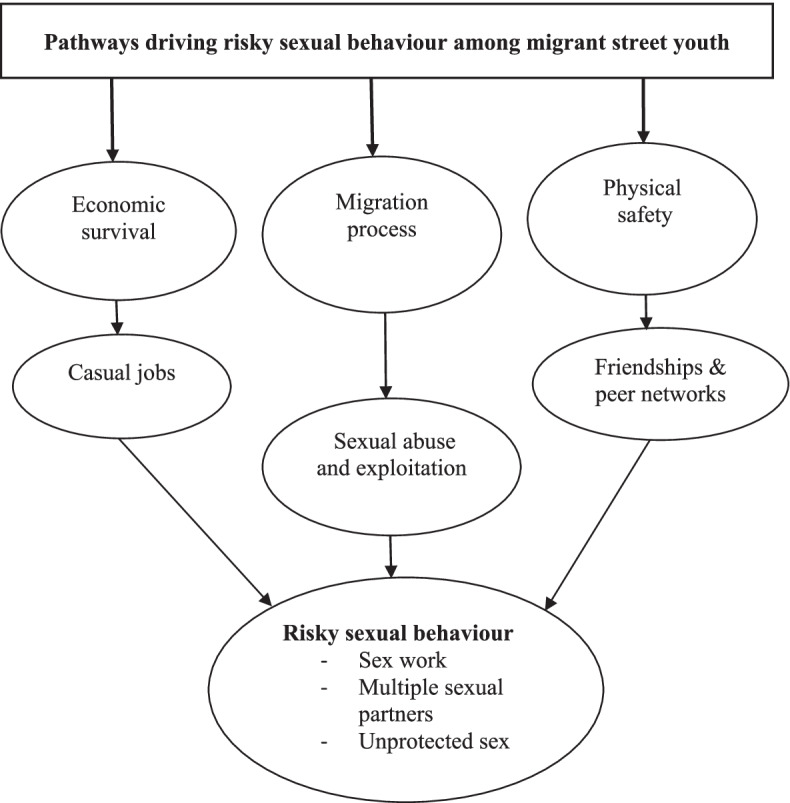
Study thematic framework

To enhance the quality of voice recording and participant’s privacy, the interviews were conducted in quieter spaces along streets which were devoid of other street youth. All the interviews were voice recorded with permission from the participants, then transcribed and translated into English. Data were analysed thematically using an inductive approach and the Dedoose software was used for coding and analysis [21, 22]. The inductive approach was considered appropriate because the research team had no prior hypothesis in mind. Also, this research aimed at generating a new theory based on the data collected, as summarised in the study framework in Fig. 1. The inductive approach typically included regular meetings among the coders to discuss procedures for assigning codes to data segments. Coding was performed by the first and second authors and two of the research assistants. To improve reliability, differences in the coding process were resolved and a comparison of codes was assigned to selected transcripts. Verbatim quotes are directly cited in the results [23]. On average, the FGDs and IDIs lasted 90 and 50 min (excluding the consent process), respectively. Access to the migrant street youth was made possible through the pre-survey mobilisation exercise and the assistance of the local leaders, social workers and guardians/caretakers of street youth. Ethical clearance was granted by the Makerere University School of Social Sciences Research and Ethics Committee (Ref. MAKSS REC 12.18.389) and the Uganda National Council for Science and Technology (Ref. HS348ES). Each FGD participant received a simple snack during the discussions while UGX 2000 (USD 0.57) was provided to both IDI and FGD participants as compensation for their participation in the study.

## Results

The study results can be summarised using the thematic framework in Fig. 1 that was generated using the findings of the FGDs and IDIs. The thematic framework in Fig. 1 suggests three primary pathways as drivers of risky sexual behaviour among migrant street youth during their movement, arrival and early integration into the city spaces: 1) the rural–urban migration process itself, through sexual exploitation and violence of street youth, especially the girls; 2) economic survival through engaging in casual jobs and sex work; and 3) personal physical safety sought through peer networks and multiple sexual partners, deemed an adaptation to the new urban street life.

### Rural–urban migration

Findings from the IDIs and FGDs with the migrant street youth showed that many young people living on the streets of Kampala travelled by road from their home districts to the city, mainly on their own. Some street youth travelled in the company of relatives and friends. They commonly used commuter taxis (*kamunyes or matatus*), buses and trucks that transport goods to and from the city. During movement, many street youth reported that they made several stops along the road before their final arrival in the city. Occasionally, sexual abuse and exploitation were considered payment for their transport, accommodation and food during the journey.

‘Some girls who did not have money to give to the transporters, sometimes had to offer sex in exchange for transport or accommodation’ [FGD, female street youth, *Ngakarimajong*].

Many of the incidences of sexual abuse occurred at the rest areas, some of which were sex work hotspots. It is at these rest areas that some street youth, especially the girls, were sexually abused. Experiences narrated by the IDI participants revealed that street girls were more prone to sexual abuse (rape or forced sex or sexual favours for food or shelter or transport) during movement than street boys. Some street girls were raped by the transporters while some male drivers had sexual intercourse with male street youth. The street youth expressed that some of the experiences they encountered along the journey were traumatic. One IDI participant had this to say:

‘One of my closest friends from Lira district, on her way to Kampala, was raped by men at a place called Karuma [a major stopover located about 200 kilometres from Kampala]. She got HIV and later she died’ [IDI, female street youth, *Luganda*].

Those migrating to the city for the first time ended up in the hands of wrong 'friends' – often fellow street youth or city adult residents who took on a caregiver’s role by assisting them with shelter or accommodation but had hidden intentions of sexual exploitation. Participants of the IDIs revealed that some fellow street youth and the purported caregivers within the urban spaces also ended up abusing them.

‘Yes, some of the street girls are just forced into sex by men. And there is nothing you can do because if you reject, the men will first beat you until you have given in for sex’ [IDI, female street youth, *Luganda*].

During the FGDs, it was mentioned that some of the street youth who travelled with their sexual partners or friends ended up losing their relationships along the journey and consequently, established new relationships with other people whom they met along the way. As a result, some of the female street youth became pregnant while some male counterparts also impregnated other women. This situation traumatised both female and male street youth as these pregnancies were unplanned. While in the city, some street youth who frequently changed places of stay found it difficult to maintain their sexual partners and hence established new sexual relationships.

‘When you arrive in the city for the first time, you don’t have where to start. So, you end up being picked up by someone to take care of you … At times, the relationship can result in pregnancy and infections when you didn’t expect it. Then, the man disappears, and you are left on your own to care for the pregnancy’ [IDI female street youth, *Luganda*].

### Personal physical safety

Upon arrival and during early integration into the city, personal physical safety became a primary concern for the street youth in the new urban environment. To improve their physical safety, many of the newcomers resorted to staying in small groups of about 5–8 people, often grouped based on gender, ethnicity and district of origin. The female street youth resorted to sleeping in guarded places as a means of securing personal physical safety. Many street youth considered their places of stay to be very unsafe especially at night, a situation that caused them to be in a state of panic and want to move to more secure and safer places.

‘We sleep in shifts such that when we notice danger, we can relocate to another street or a place to sleep where it is safer’ [IDI, male street youth, *Ngakarimajong*].

During the IDIs, many street youth reported that people who supported them in the form of providing temporary shelters at night occasionally abused them sexually. The situation was worse for the street girls who did not have money to pay for accommodation. Some street girls were forced into sex by the private security guards of shopping malls and markets who offered them a place or a veranda to sleep at night.

‘If you do not have money to pay for a place to sleep, sometimes they [can] forcefully sleep with you in exchange for space’ [IDI, female street youth, *Luganda*].

The street children and youth lived in rooms and shelters that were occupied either by one gender or shared between females and males. Such living arrangements were found to catalyse early sexual initiation practices, with the younger girls and boys being initiated by their older peers. For instance, during the FGDs, some street youth mentioned that their older counterparts with whom they stayed engaged in sexual activities in their presence and with no respect to privacy. Participants reported that the acts of sexual abuse (rape) were more common among the newcomers compared to those who had stayed a little longer in the city and acquired more stable social networks and shelter, implying that duration of stay may have an influence on sexual behaviour over time.

On the other hand, friendships and social networks were found to be a source of peer pressure to encourage migrant street youth, especially the newcomers, into risky sexual behaviour.

‘…but mostly it is all about group influence; so, when you see others [street youth] going for sex work at places such as Kabalagala, you end up into sex work’ [FGD, male street youth, *Ngakarimajong*].

In addition, some street youth reported having engaged in risky sexual activities partly due to their inability to resist sex due to poor shelter conditions, as some street female youth shared the same rooms or shelters with the opposite sex. They reported that use of alcohol and other illicit drugs was common in the places where they stayed, and this influenced some of them to engage in sexual activities without self-control. One of the FGD female participants remarked:

‘I got drunk, and I went in for sex with a man without a condom’ [FGD female street youth, *Luganda*].

### Economic survival on the street

In addition to personal physical safety, street youth described economic survival as another factor that drove them to risky sexual behaviour. During the interviews, many street youth said that they migrated to Kampala in search of greener pastures and with the hope of a better life. On arrival in the city, their expectations for jobs were either completely unmet or not fully met, forcing them into risky sexual behaviour as a means of survival. Most of the street youth were involved in casual jobs, such as working as restaurant attendants, and vending goods on the streets for survival. Sometimes, some street girls had to offer sex in exchange for the casual jobs in restaurants and bars where they served as attendants. These places of work exposed them to sex work as complementary income to what they earned from the causal jobs themselves.

‘…when you fail to get a well-paying job, you find yourself doing bad things like sleeping [having sex] to get what to eat and send some money back home’ [FGD, female street youth, *Ngakarimajong*].

During the interviews, many street youth frequently mentioned that they had no intentions of soliciting any sexual favours from anyone upon their arrival in the city. Rather, it was the immense hardships they faced in the new city environment, including their inability to find decent work, food, a place to sleep and to meet other basic needs that pushed them into risky sexual behaviour. In addition to meeting their personal needs, some street youth had obligations of sending financial support back home to their families and children left behind.

‘We have a lot of needs. At times, we don’t get the money that will sustain us. So, you end up going in for a man who can give you at least UGX 5000 [USD 1.4] which can push you through the day’ [FGD female street youth, *Luganda*].

The enormous needs of the migrant street youth created an urgency to earn extra income daily, with the returns from sex work thought to be more lucrative compared to the other livelihood options. The sense of urgency seemed to vary by gender, in which most street girls were under more pressure to meet their immediate basic needs, such as food and accommodation within the city spaces, than the street boys.

Some street youth believed that some of their peers migrated to the city out of frustration regarding the poverty in the rural areas and their expectations were sometimes not met. Many street youth explained that the females were more vulnerable to engaging in sex work than the males. They believed that the male street youth had more options to make money compared to their female counterparts. The issue of the affordability of condoms emerged with some street youth attributing their powerlessness to resist unprotected sex to their inability to buy condoms. One IDI participant noted that:

‘As a girl, you leave your home [district] with the expectation of getting a job in the city’ [IDI female street youth, *Ngakarimajong*].

## Discussion

This qualitative enquiry explored pathways that drive risky sexual behaviour among rural–urban migrant street youth in Kampala, Uganda within the context of rural–urban migration. Our findings showed that the rural–urban migration process exposed some street youth to different forms of sexual abuse such as rape and sex in return for favours. Upon arrival and during early integration in the city, street youth engagement in sex work, multiple sexual partnerships and unprotected sex was driven by a desire for both personal physical safety and the ability to survive and adapt to the new urban environment and street life.

The study findings show that the rural–urban migration process increased migrant street youth’s vulnerability to risky sexual behaviour, especially sexual abuse and exploitation. The risky sexual behaviours experienced by the migrant street youth are similar to those reported among immigrant youth in South Africa and Europe [9, 24, 25]. Despite the similarities, the social and economic contexts of the studies are different; hence the approaches to tackling the risky sexual behaviour could be different. The nature and gravity of the sexual risks could be more concerning for the street girls in Kampala than the adult female immigrants in the European study who may have better access to social services [25].

Personal physical safety upon arrival in the city was another pathway that pushed the migrant street youth into risky sexual behaviour, mainly through social networks. The study revealed that staying together in small ethnic-based groupings partly improved personal physical safety and protection from potential sexual abuse. This finding may imply that the city spaces offer migrant street youth less access to social safety and support. On the other hand, our study revealed that the lack of personal physical safety due to inappropriate shelter or living conditions exposed young street girls and boys to sexual initiation practices by their older peers. This finding contrasts with earlier studies in which social networks have been found to be protective against risky sexual behaviour [22]. The sexual exploitation of street girls by their adult male peers, the private security guards and sham landlords or caretakers who offer temporary shelters to street youth, needs to be addressed and perpetrators punished through better enforcement of the Uganda Child Protection Act (2016).

Economic survival as a pathway to risky sexual behaviour among migrant street youth was another major finding of this study. Survival through sex work and multiple sexual partners could be a form of adaptation to the challenging city environment in which the street youth must survive to meet their basic needs. The places where street youth find work, especially the streets, bars and restaurants, have been found to be sex work hotspots and are housing opportunities affordable to the urban poor, which may predispose them to sex work as an occupational risk. Elsewhere, street youth have been found to engage in exploitative activities to survive, including maximising income from sex work through unprotected sex [23, 26]. While economic survival through sex work helped to address the immediate needs of the street youth, the associated risks may far outweigh the economic benefits derived from sex work. Therefore, interventions to address the basic needs of street youth, such as food, shelter and finding decent work through vocational skills training as well as establishing condom promotion programmes could help reduce their vulnerability to risky sexual behaviour. These interventions could be modelled on the already existing peer social networks and peer education approaches.

Despite the challenges migrant street youth faced in the city, it should be acknowledged that the livelihoods and healthcare system may still be better in cities than in the rural areas where access to social services is limited. During the interviews, some migrant street youth expressed that they were not fully involved in the planning and implementation of the health services. They demanded that KCCA and service providers such as Non-Governmental Organisations (NGOs) should deliver tailored SRH information and services using appropriate local languages and within the urban spaces where they live or work.

Similarly, multi-level interventions to reduce sexual risks should not only focus on preventing and mitigating risky sexual behaviour, but also ensure that the migration process is humane and safe for the young people. Some of the essential elements to make migration humane could include adherence to human rights standards, partnerships and involvement of all sectors of government in migration policy processes and enhancement of socio-economic wellbeing of the migrants and society [27].

While this study was limited to exploring the pathways that drive risky sexual behaviour among migrant street youth, the observed associations are based on perceptions and experiences and hence their actual sexual behaviour may not have been fully captured as they may have been shy to disclose. Stakeholders such as the government and NGO officials and healthcare service providers were excluded from the study because the research team believed that their perceptions may not truly reflect the street youth’s actual sexual behaviour and migration narratives. The research team acknowledges that some drivers of risky sexual behaviour may be explained by cultural, religious and economic characteristics of the street youth which this study did not pay attention to. The study could have also suffered from recall bias as some street youth may not have fully recollected their past experiences. Although the study thematic framework shows a linear relationship between three pathways that drive risky sexual behaviour, it is also possible that the variables across the pathways may be interconnected. This study serves as a useful point of departure for future research to examine the multiple and interconnected pathways that may influence risky sexual behaviour among migrant street youth. Nonetheless, the study demonstrates the important role the rural–urban migration process, physical safety and economic survival play in shaping migrant street youth’s risky sexual behaviour along the mobility continuum.

## Conclusions

The study findings provide useful insights into the importance of the rural–urban migration process, personal physical safety and economic survival as pathways driving risky sexual behaviour among migrant street youth in Kampala. In addition, the findings call for the need for the Kampala Capital City Authority (KCCA), Ministry of Health and other stakeholders to design policies and strategies to improve sexual and reproductive health, housing conditions and protection from sexual exploitation and abuse and safety of urban street youth, including the recognition of street children and youth as key and vulnerable populations in national and sub-national policies and plans. Future research to explore the associations between rural–urban migration, sexual behaviour and healthy SRH choices among street youth could provide a comprehensive picture on this important topic.

## Data Availability

The datasets used and/or analysed during the current study are available from the corresponding author on reasonable request.
